# Genome-Wide Identification and Characterization of the *PERK* Gene Family in *Gossypium hirsutum* Reveals Gene Duplication and Functional Divergence

**DOI:** 10.3390/ijms20071750

**Published:** 2019-04-09

**Authors:** Ghulam Qanmber, Ji Liu, Daoqian Yu, Zhao Liu, Lili Lu, Huijuan Mo, Shuya Ma, Zhi Wang, Zuoren Yang

**Affiliations:** 1 State Key Laboratory of Cotton Biology, Key Laboratory of Biological and Genetic Breeding of Cotton, Institute of Cotton Research, Chinese Academy of Agricultural Sciences, Anyang 455000, China; gqkhan12@gmail.com (G.Q.); liuji@caas.cn (J.L.); yudaoqian88@163.com (D.Y.); liuzhaocaas@163.com (Z.L.); biolll@126.com (L.L.); mohuijuan86@163.com (H.M.); msymsy89@126.com (S.M.); 2 Zhengzhou Reseach Base, State Key Laboratory of Cotton Biology, Zhengzhou University, Zhengzhou 450000, China

**Keywords:** *G. hirsutum*, *GhPERK*, sequence logos, phylogenetic analysis, *cis*-elements, gene duplication, abiotic stress, phytohormone stress

## Abstract

Proline-rich extensin-like receptor kinases (*PERKs*) are an important class of receptor kinases in plants. Receptor kinases comprise large gene families in many plant species, including the 15 *PERK* genes in *Arabidopsis*. At present, there is no comprehensive published study of *PERK* genes in *G. hirsutum*. Our study identified 33 *PERK* genes in *G. hirsutum*. Phylogenetic analysis of conserved PERK protein sequences from 15 plant species grouped them into four well defined clades. The *GhPERK* gene family is an evolutionarily advanced gene family that lost its introns over time. Several *cis*-elements were identified in the promoter regions of the *GhPERK* genes that are important in regulating growth, development, light responses and the response to several stresses. In addition, we found evidence for gene loss or addition through segmental or whole genome duplication in cotton. Gene duplication and synteny analysis identified 149 orthologous/paralogous gene pairs. Ka/Ks values show that most *GhPERK* genes experienced strong purifying selection during the rapid evolution of the gene family. *GhPERK* genes showed high expression levels in leaves and during ovule development. Furthermore, the expression of *GhPERK* genes can be regulated by abiotic stresses and phytohormone treatments. Additionally, *PERK* genes could be involved in several molecular, biological and physiological processes that might be the result of functional divergence.

## 1. Introduction

Receptor kinases comprise large gene families in many plant species, including rice and *Arabidopsis*. The *Arabidopsis* receptor kinase family contains approximately 600 members and their homologs have been investigated in ~20 different species [1,2,3]. The biological functions of most of these predicted receptor kinase proteins are unknown. However, receptor kinases are known to play essential roles in signal transduction, the response to external challenges in an ever-changing environment, hormone response pathways, plant growth and development, self-incompatibility, cell differentiation and symbiosis and pathogen recognition. Some studies have identified ligands that activate these receptor kinases and other signaling components as well [4,5]. Receptor kinases bind to a diverse array of molecules including steroids, polypeptides, carbohydrates and cell wall components, depending on their extracellular domain. Receptor kinases transduce signals across the plasma membrane via different signaling complexes found in eukaryotic cells other than plants and likely developed early during the evolution of multicellular organisms [2,5].

Previously, receptor kinases were classified into many distinct classes on the basis of motifs in their extracellular domains [5,6]. For example, the leucine-rich repeat (LRR) receptor kinase family is the largest class of *Arabidopsis* receptor kinases, including *BRI1* and *BAK1*, which are involved in brassinosteroid (BR) perception [7,8,9]. Among the different classes of receptor kinases, gene duplications leading toward functional redundancy have also been observed [10]; for example, the *CLV1* and *ERECTA* receptor kinases are evidence that functional redundancy exists [11,12,13]. Proline-rich extensin-like receptor kinases (*PERKs*) are an important class of receptor kinases. The *Arabidopsis PERK* family is similar to *Brassica napus PERK1* and shares maximum sequence identity. In the *Arabidopsis* genome, a total of 15 *PERK* genes have been identified; however, their biological functions are largely unknown [14,15]. *PERK1* is localized to the plasma membrane as predicted for a receptor kinase and the gene is induced rapidly in response to wounding in *Arabidopsis* [14]. Furthermore, *PERK4* is a novel regulator of Ca^2+^ signaling that also mediates the early events leading up to the abscisic acid response in root tip growth [16,17].

Plants face continuously changing environmental conditions during growth and development. Among these environmental challenges, abiotic and hormonal stresses are the crucial factors that affect plant growth and biological yield. Cotton is one of the leading fiber crops cultivated worldwide [18]. Various abiotic stresses and hormonal homeostasis, such as BR, play essential roles in its development and the quality of the cotton fiber [19,20]. Also, advancements in cotton genome sequencing [21,22,23,24,25,26] have made it possible to conduct a comprehensive investigation of cotton genes. In our study, we performed a systematic analysis of 207 *PERK* genes in 15 different plant species, including 33 *PERK* genes in *G. hirsutum*. Next, sequence logos, phylogenetic analysis, biophysical properties, exon/intron and protein motif distribution, promoter *cis*-element analysis, chromosomal distribution, gene duplication, synteny analysis and Ka/Ks values were also determined. Furthermore, tissue-specific expression patterns, abiotic stress responses and functional roles with respect to BR signaling and other phytohormones were investigated for the *G. hirsutum PERK* genes. The present study will enable a detailed understanding of the molecular and biological functions of *PERK* genes in cotton.

## 2. Results

### 2.1. Identification of PERKs

In this study, we used various *in silico* approaches to identify a total of 207 PERK gene family members in 15 different plant species following confirmation with different tools such as PROSITE (http://prosite.expasy.org/), SMART (http://smart.embl-heidelberg.de/) and InterProscan 63.0 (http://www.ebi.ac.uk/interpro/) (Table S1). The *PERK* gene family includes 15 genes from *Arabidopsis*, 15 from *G. arboreum*, 33 from *G. hirsutum*, 16 from *G. raimondii*, 16 from *G. max*, 8 from *O. sativa*, 14 from *P. trichocarpa*, 15 from *S. bicolor*, 9 from *T. cacao*, 23 from *Z. mays*, 12 from *A. comosus*, 10 from *C. reinhardtii*, 5 from *P. patens*, 10 from *S. moellendorffii* and 6 genes from *P. taeda*. We found that almost all selected plants have at least five *PERK* genes with *G. hirsutum* having the highest number (33) of *PERK* genes and *P. patens* having only five genes, indicating that *PERK* genes were subjected to a large scale expansion in higher plants. Because our main interest is in upland cotton, *G. hirsutum*, we compared the retrieved sequences of two different sequenced genomes of upland cotton including BJI and NAU and found no differences among the candidate genes. For the following analyses we used the gene sequences retrieved from the NAU genome sequence database. Here, allotetraploid upland cotton had more than double the number of *PERK* genes compared to diploids cotton species such as *G. arboreum* and *G. raimondii*, illustrating the effect of polyploidy on the *PERK* gene family in *G. hirsutum* which is derived from the hybridization of two progenitor species resembling the diploids *G. arboreum* and *G. raimondii*.

We next determined the biophysical properties of the *GhPERK* family members including locus ID, the corresponding chromosome, start and end points, strand polarity, gene length (bp), CDS length (bp), protein length (aa), predicted protein molecular weights (MW) and isoelectric points (pl), predicted masses and predicted cellular localization. We found that 14 *GhPERK* genes originated from the At sub-genome, 15 from the Dt sub-genome and four were from scaffolds of the *G. hirsutum* genome. Predicted protein lengths range from 178-884 aa, with molecular weights (MW) of 19,755.66–93,594.32 Da for *GhPERK7* and *GhPERK14*, respectively. Moreover, the cellular localizations of all GhPERK proteins were related to the plasma membrane. All other estimated biophysical properties are shown in Table S2.

### 2.2. Sequence Logos and Phylogenetic Analysis

**Figure 1:**
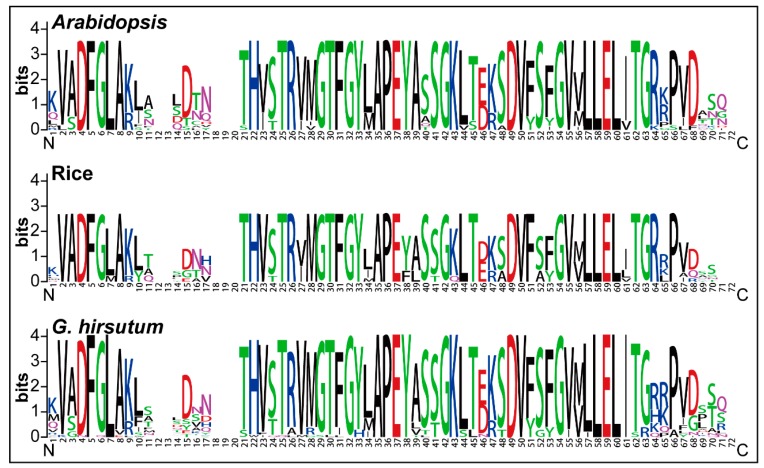
Sequence logos of conserved amino acid residues in *Arabidopsis*, Rice and *G. hirsutum*.

To check whether the PERK family proteins were conserved during evolution, we generated the sequence logos of the conserved amino acid residues in *Arabidopsis*, rice and upland cotton *G. hirsutum* (Figure 1). We found that the sequence logos among the three species were highly conserved across the N and C termini such as D [4], F [5], H [22], T [25], G [27], T [26], G [28], A [29], P [30], E [31], G [32], D [33], V [34], G [35], L [36], E [37], L [38] and so forth. Also, no compositional bias of any specific pattern of conserved amino acid residues was observed in sequence logos of *Arabidopsis*, rice and *G. hirsutum*. As sequence logos provide a more precise description of sequence similarity than consensus sequences, significant features of the alignment, patterns in sequence conservation and assist to discover and analyze those patterns [39]. In this regard, PERK protein sequence logos in *Arabidopsis*, rice and *G. hirsutum* will assist to discover, analyze and evaluate the pattern of PERK protein sequence conservation in other plant species.

**Figure 2:**
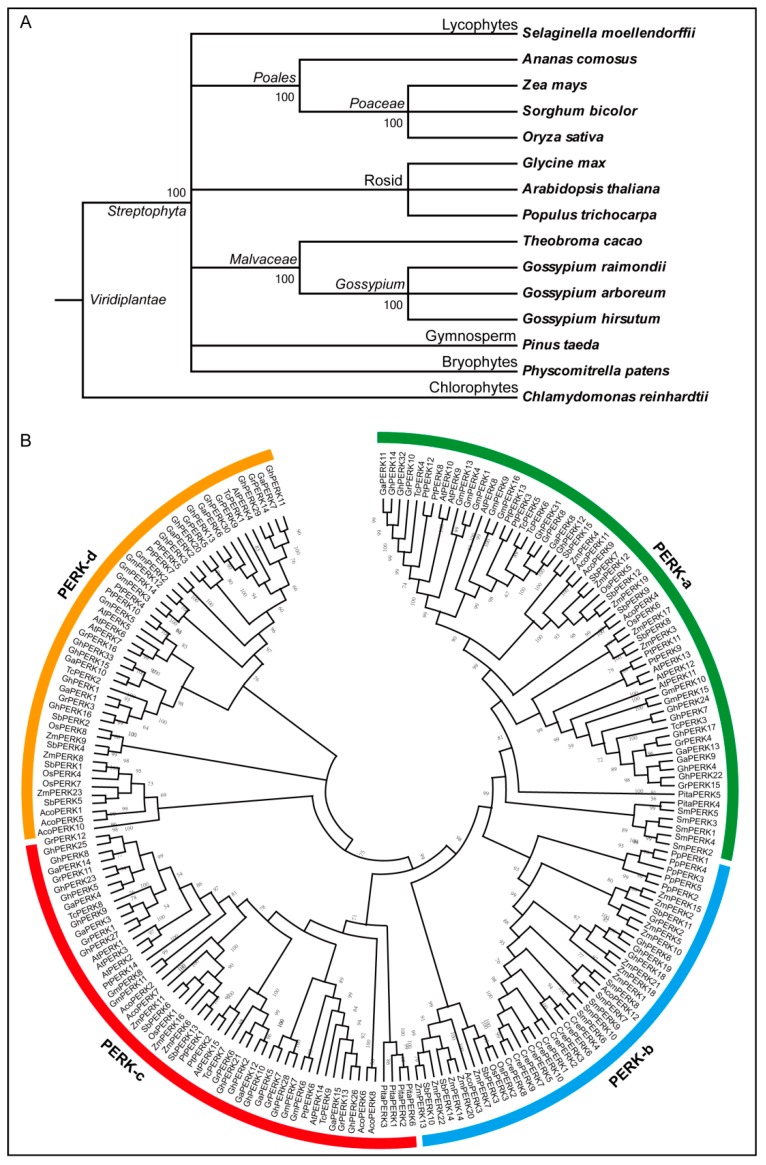
Phylogenetic analysis of *PERK* genes from 15 plant species. (**A**) The phylostratum analysis of *PERK* gene family. (**B**) Phylogenetic tree divided all 207 *PERK* genes into four groups from PERK-a to PERK-d. Prefixes such as At, Ga, Gh, Gr, Gm, Pt, Tc, Os, Zm, Sb, Aco, Cre, Pp, Sm and Pita were used before the names of *A. thaliana, G. arboreum, G. hirsutum G. raimondii, G. max, P. trichocarpa, T. cacao*, *O. sativa, Z. mays*, *S. bicolor*, *A. comosus*, *C. reinhardtii*, *P. patens*, *S. moellendorffii* and *P. taeda PERK* genes respectively. Bootstrap values were also mentioned near node of each branch.

The phylostratum analysis of *PERK* gene family identified the earliest plant lineage as *PERK* genes were present in *C. reinhardtii* (chlorophyte), an earliest plant lineage (Figure 2A). Further, the *PERK* genes were present in *A. comosus* (angiosperm), *P. taeda* (gymnosperm), *P. patens* (bryophytes), *S. moellendorffii* (lycophytes), dicots (*A. thaliana, G. arboreum, G. hirsutum, G. raimondii, G. max, P. trichocarpa* and *T. cacao*) and monocots (*O. sativa, S. bicolor* and *Z. mays*). These results indicated that *PERK* genes were originated from early land plants phylostratum and potential orthologous genes of *PERK* are present throughout pant kingdom. Next to construct the evolutionary relationships among the 207 *PERK* genes from 15 different plant species including *A. thaliana, G. arboreum, G. hirsutum, G. raimondii, G. max, P. trichocarpa*, *T. cacao*, *O. sativa, S. bicolor*, *Z. mays*, *A. comosus*, *C. reinhardtii*, *P. patens*, *S. moellendorffii* and *P. taeda*, a Maximum likelihood (ML) tree was generated. Phylogenetic analysis grouped all PERK proteins into four clades (PERKa-d) on the basis of their sequence homologies (Figure 2B).

In the phylogenetic analysis, the PERK-a clade contained 62 members, PERK-b contained 44 members, PERK-c contained 53 members and PERK-d contained 48 members of the PERK protein family. PERK-a clade contains genes from *S. moellendorffii* (lycophytes), *P. taeda* (gymnosperm), *A. comosus* (angiosperm), dicots and monocots except *C. reinhardtii* (chlorophyte) and *P. patens* (bryophytes), indicated the evolution of these *PERK* genes after the split of lycophytes, chlorophyte and bryophytes. While PERK-b clade contains *PERK* genes from all plant species except *P. taeda*. Similarly, PERK-c clade lacks the *PERK* family members from *S. moellendorffii*, *C. reinhardtii* and *P. patens*. While PERK-d clade lacks the *PERK* genes from *S. moellendorffii*, *C. reinhardtii*, *P. patens* and *P. taeda*. Surprisingly, *PERK* genes from *C. reinhardtii* were only present in clade PERK-b. Interestingly, *PERK* genes from dicot and monocot plant species were randomly distributed to all four clades. Moreover, *PERK* genes from *G. hirsutum* were present in almost all clades with PERK-c having highest number (11) of *GhPERK* genes and PERK-b having only three *GhPERK* genes. These results demonstrated that PERK-b clade is an ancient group of *PERK* genes having *PERK* gene family members from almost all plant species except *P. taeda*. Further, the phylogenetic analysis indicated that *G. hirsutum* experienced significant gene family expansion, because it has almost double the number of *PERK* genes compared to the other plant species except *Z. mays*. At least eight pairs or triplets of GhPERK proteins cluster together in the phylogenetic tree, which can be interpreted as evidence of gene duplication. All species except for *G. raimondii* and *S. bicolor* had gene pairs derived from the same node, demonstrating that the *PERK* genes in all species included in our study have experienced gene duplication events that resulted in *PERK* gene family expansion. However, gene duplication among the different groups varies for the different species. These results indicate that gene duplication is the main contributor toward *PERK* gene family expansion in all plant species. These results could be considered as evidence for lineage-specific expansion of the *PERK* genes after the divergence of dicots and monocots. Further, a NJ tree was constructed for the cotton PERK proteins in order to investigate the relationship between the common ancestors of diploid (*G. arboreum* and *G. raimondii*) and allotetraploid cottons (*G. hirsutum*) (Figure S1). The tree grouped all cotton PERK proteins into six clearly-defined clades, each of which contains proteins from both diploid and allotetraploid cotton species. The clusters provide evidence confirming that *G. hirsutum* is the result of hybridization between the two diploid cotton species *G. arboreum* and *G. raimondii*.

### 2.3. Exon/Intron, Protein Motif and cis-element Analysis

It has been previously reported that the exon/intron distribution pattern in a gene is related to its biological function. Exon/intron analysis along with phylogenetic tree results showed that all *GhPERK* genes lack introns and that similar genes were clustered close to one another in the same groups (Figure S2A). Furthermore, the protein motif distribution patterns of the GhPERK proteins revealed that similar motifs had conserved distribution patterns, even in the case of GhPERK19, GhPERK18 and GhPERK6 that lacked certain motifs (Figure S2B). In fact, we found that all *GhPERK* genes showed conserved patterns of gene structure (exon/intron) and protein motif distribution for the At and Dt sub-genomes even though gene duplication occurred long ago during evolution.

Because gene expression is regulated by promoters and by the binding of transcription factors to *cis*-elements located in upstream regions, *cis*-element analysis is important to understanding gene regulation and function [27,40]. We identified *cis*-elements in the promoters of the *GhPERK* genes and categorized them based on their functional relevance (Table S3). The identified *cis*-elements were classified with respect to their functions in growth and development and light and stress responses. All of the *GhPERK* genes had various *cis*-elements for different responses such as the GCN4 and Skn-1 motifs (endosperm expression), the as-2-box (shoot-specific expression), the 3-AF1 binding site, AC-II, the CCGTCC-box, Sp1 and circadian for growth and development, ACE, AE-box, ATCT-motif, Box 4, Box I, Box II, G-box, GA-motif, GAG-motif, GATA-motif, GC-motif, GT1-motif, I-box, L-box, Sp1, TCT-motif and chs-CMA1a for light response and MBS and TC-rich repeats (For stress and defense), LTR (for low temperature), Box-W1 (fungal elicitor), ARE, CGTCA-motif, GARE-motif, HSE, TCA-element, TCCACCT-motif, TGACG-motif, TGA-element and TCCC-motif for different stress responses. Additional *cis*-elements that regulate responses to different hormones such as auxin (TGA-element and AuxRE-core), salicylic acid (TCA-element and SARE), gibberellin (TATC-box, P-box and GARE-motif), MeJA (CGTCA-motif and TGACG-motif) and ethylene (ERE-box) were also identified in the promoter regions of many *GhPERK* genes.

### 2.4. Chromosomal Distribution, Gene Duplication and Synteny Analysis

All identified *GhPERK* genes were mapped to their corresponding chromosomal loci. The results showed that 14 genes mapped to At sub-genome chromosomes, 15 to Dt sub-genome chromosomes and four to scaffolds (Figure S3). Most of the chromosomes contained only a single *GhPERK* gene, although D01 contained three genes and there were no *GhPERK* genes identified on chromosomes D02, A03, A09 and its homolog D09. The absence of any *PERK* genes on A03 is supported by a previous study in which a translocation between chromosomes A02 and A03 was reported. Furthermore, the absence and uneven distribution of *GhPERK* genes on some At and Dt sub-genome chromosomes and scaffolds indicates that gene loss may have occurred during evolution but an incomplete genome assembly could also be a factor.

**Figure 3:**
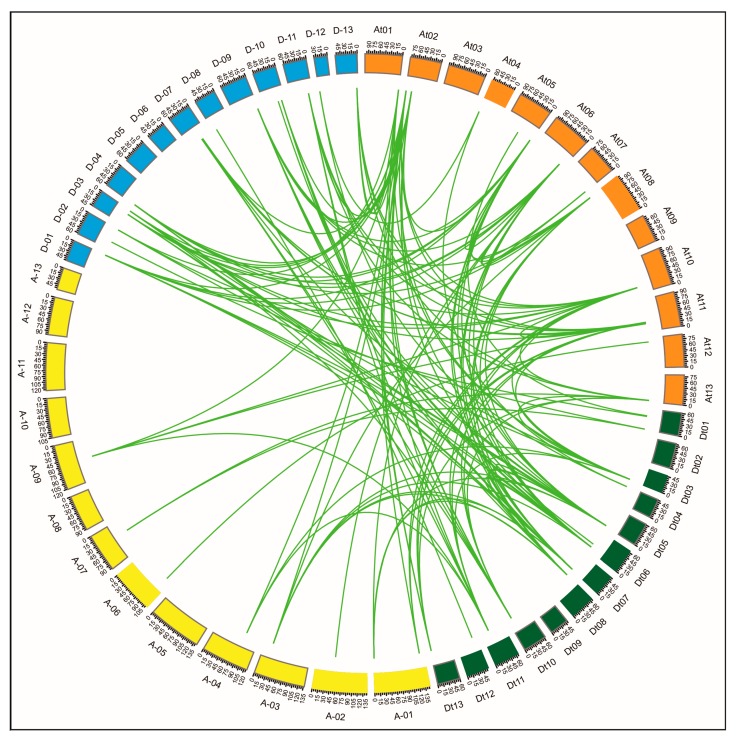
Gene duplication among cotton *PERK* genes including *G. hirsutum* (At and Dt sub-genome), *G. arboreum* (A-genome) and *G. raimondii* (D-genome). Green lines exhibited orthologous/paralogous pairs. At01-At13 (orange blocks) indicated chromosomes of At sub-genome and Dt01-Dt13 (dark green blocks) represented chromosomes of Dt sub-genome. Similarly, A-01 to A-13 (yellow blocks) and D-01 to D-13 (light blue blocks) represented *G. arboreum* and *G. raimondii* chromosomes, respectively.

To study the locus relationships among the *GhPERK* genes, we identified the orthologous/paralogous gene pairs for the At and Dt sub-genomes of upland cotton (*G. hirsutum*). Upland cotton is an allotetraploid crop that is a model species for the study of natural polyploidy [26]. Synteny analysis indicated that several gene loci are highly conserved between the At and Dt sub-genomes of upland cotton (Figure 3). As described above, upland cotton resulted from the hybridization between the ancestors of the diploid species *G. arboreum* and *G. raimondii* [28,41]. Similar to previous findings, our study also demonstrated that the At as well as the Dt sub-genomes have orthologous in the A (*G. arboreum*) or D (*G. raimondii*) genomes and the observation that the genes from any one from these four genomes (At, Dt, A and D) have the corresponding orthologous from the other three genomes provides evidence that cotton *PERK* genes may not have experienced genomic arrangements during polyploidization. A total of 149 orthologous/paralogous gene pairs were identified, of which 10 pairs were attributed to segmental duplication to form paralogous gene pairs within the At or Dt sub-genomes (Table S4). From these results we assumed that paralogous gene pairs were mainly contributed by segmental or whole genome duplication prior to polyploidization. We also identified these duplicated gene pairs in diploid cottons and their presence demonstrated that gene duplication occurred before the hybridization and formation of the allotetraploid species *G. hirsutum* (Figure 3 and Table S4).

During evolution, duplicated gene pairs can experience functional divergence resulting in neo-functionalization, sub-functionalization, or non-functionalization [42]. To determine the nature and extent of selection pressure on these duplicated gene pairs, we calculated their Ka/Ks values (Table S4). The Non-synonymous (Ka) and synonymous (Ks) divergence values showed that for 129 duplicated gene pairs, the Ka/Ks ratios were <0.5, while for another 17 pairs, Ka/Ks was between 0.5 and 1.0. The only exception was that the Ka/Ks ratio for three gene pairs was >1.0, illustrating that cotton the *PERK* genes have undergone strong purifying selection, since Ka/Ks was <1.0 for 146 out of 149 duplicated gene pairs and only three duplicated gene pairs showed positive selection pressure.

### 2.5. Tissue-Specific Gene Expression Patterns and Abiotic Stress Responses

**Figure 4:**
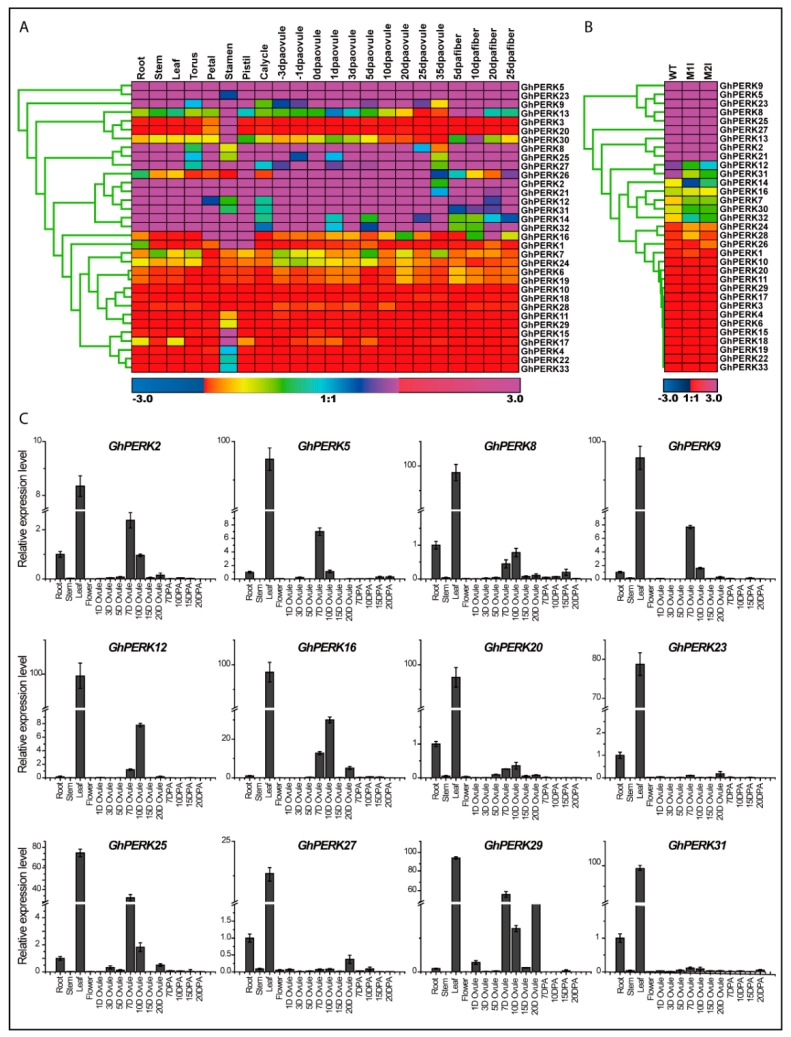
Relative expression level of *GhPERK* genes in different tissues of cotton plant. (**A**) Heat map of 33 *GhPERK* genes in 22 different tissues. Data was obtained from publicly available transcriptomic data and color bar (down) represented expression level. (**B**) Heat map of *GhPERK* genes relative expression in two fuzzless/lintless mutants (*M1l and M2l*) in comparison to WT plants. Data was obtained from published RNA-seq data and color bar (down) indicated the value of expression level. (**C**) Relative expression level by qRT-PCR analysis of selected *GhPERK* genes. Error bars indicated the standard deviations (SD) among three independent biological repeats.

Regulated gene expression is critical for normal plant growth and development. Expression patterns can provide clues into the biological functions of genes, so we examined the expression patterns of the *GhPERK* genes in different cotton tissues. First, we analyzed *GhPERK* gene expression in published transcriptomic data downloaded from NCBI (https://www.ncbi.nlm.nih.gov/pmc/articles/PMC4482290/) for 22 different tissues including roots, stems and leaves, reproductive tissues such as torus, petals, stamens, pistils, calyces and ovules sampled at -3, -1, 0, 1, 3, 5, 10, 20, 25 and 35 days post-anthesis (DPA) and fibers sampled at 5, 10, 20 and 25 DPA. Heat mapping of gene expression indicated that genes with similar expression levels clustered close together and that the expression patterns for many of the genes differed considerably in the different tissues (Figure 4A). Furthermore, because the fiber is the product harvested from cotton, we evaluated the expression of *GhPERK* genes in previously published RNA-seq data from two lintless/fuzzless mutants named *M1l* and *M2l* [43] and generated a heat map of the expression levels (Figure 4B). The map showed that expression of one gene (*GhPERK1*) was up-regulated and two genes (*GhPERK13* and *GhPERK26*) were down-regulated in the *M1l* mutant, while the three genes display the reverse expression patterns in the *M2l* mutant. However, the relative expression of 10 genes was the same in both mutants. Overall, 13 *GhPERK* genes were down- while seven *GhPERK* genes were up-regulated for both mutants, indicating both positive and negative roles in cotton fiber formation (Figure 4B and Table S5).

From here, we selected 12 of the most down-regulated genes and examined their tissue-specific expression patterns by qRT-PCR analysis in roots, stems, leaves, flowers, ovules (1, 3, 5, 7, 10, 15 and 20 DPA) and fibers (7, 10, 15 and 20 DPA). Interestingly, the results were generally similar to analyses of previously published transcriptomic and RNA-seq data, as all 12 genes were highly expressed in leaves and also during some stages of ovule development. However, all estimated *GhPERK* genes did not show any obvious contribution in fiber development and other tissues except for root by some genes (Figure 4C). Together with these results, we speculated that *GhPERK* genes played roles in leaf development as well as for ovule development with few exceptions.

**Figure 5:**
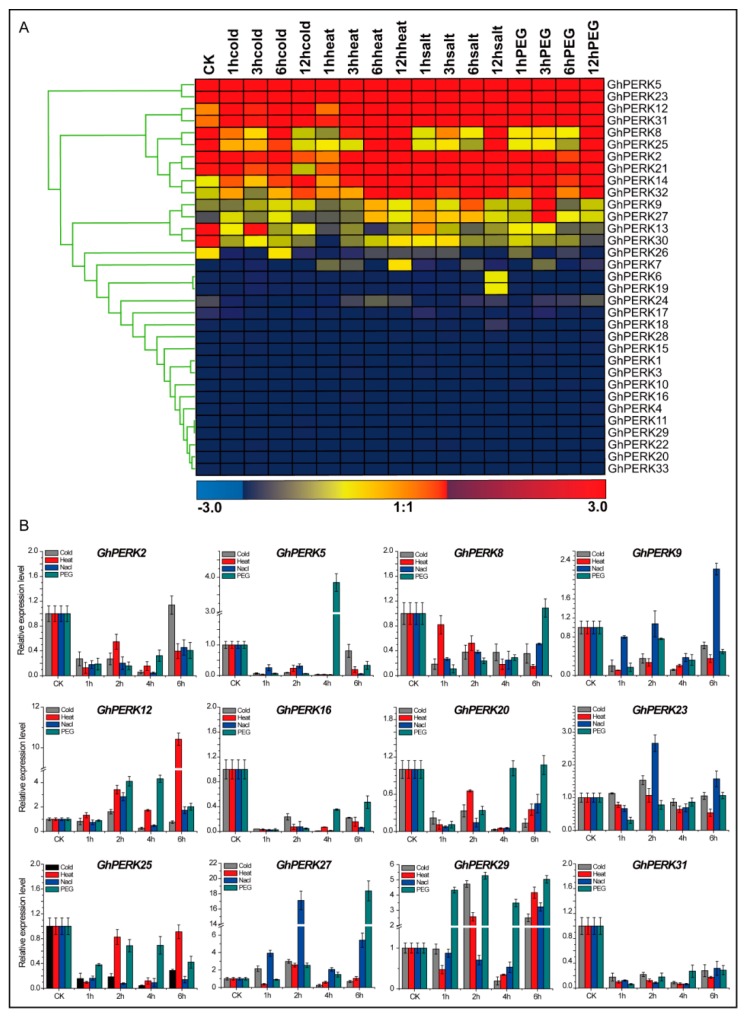
Expression analysis of *GhPERK* genes for four different abiotic stresses including cold, heat, NaCl and PEG. (**A**) Heat map of *GhPERK* genes constructed by publicly available transcriptomic data. Color bar (down) depicted the level of response for that gene. (**B**) qRT-PCR expression pattern of *GhPERK* genes under abiotic stresses. The error bars exhibits standard deviations (SD) among three independent biological repeats.

Cotton plants face several abiotic stress challenges during their growth and development; therefore, we next evaluated *GhPERK* gene expression in response to various abiotic stresses. First, we analyzed the relative expression of *GhPERK* genes in publicly-available transcriptomic data for changes related to abiotic stresses including cold, heat, salt and PEG (drought) at several time points [24]. A heat map showed that the expression of all *GhPERK* genes was up- or down-regulated under different treatments at different time points (Figure 5A). Genes displaying similar responses clustered close together. In addition, we used qRT-PCR to estimate the expression levels of selected genes in response to cold, heat, NaCl and PEG after 1, 2, 4 and 6 hours of treatment (Figure 5B). Similar to the transcriptome analysis, most of the *GhPERK* genes were found to be down-regulated at different time points for most of the abiotic stress treatments. *GhPERK12*, *GhPERK27* and *GhPERK29* expression was mostly up-regulated at different time points for all of the tested stress treatments with a few exceptions. However, the expression of *GhPERK8*, *GhPERK16*, *GhPERK20*, *GhPERK25* and *GhPERK31* was down-regulated for most or all treatments. From these results, we can assume that expression of *GhPERK* genes shows sensitivity to abiotic stresses and most genes are generally down-regulated in response to the four different abiotic stresses.

### 2.6. GhPERK Gene Expression in Response to Phytohormone Treatments

**Figure 6:**
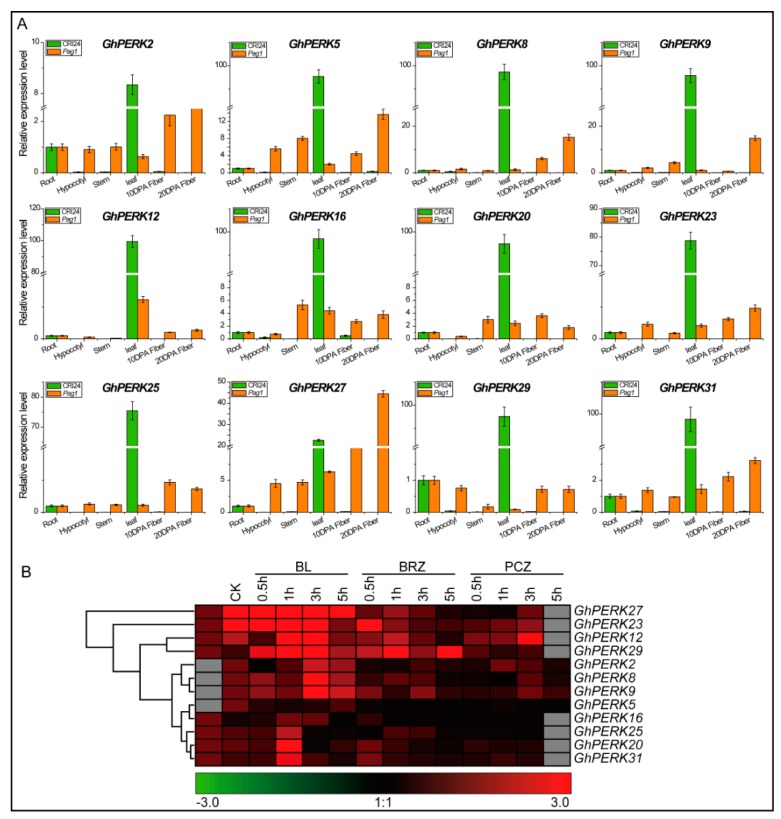
Expression pattern analysis of *GhPERK* genes for brassinosteroid (BR) hormone. (**A**) Relative expression level of *GhPERK* genes in *Pag1* (cotton brassinosteroid mutant) and CRI24 (WT) evaluated by qRT-PCR analysis. (**B**). Heat map generated by the relative expression values estimated by qRT-PCR analysis on the exposure of BL, BRZ and PCZ treatment. Error bars exhibits standard deviations (SD) among three independent biological repeats.

To investigate the roles of *GhPERK* genes in response to the phytohormone brassinosteroid (BR), first we analyzed the tissue-specific expression levels of selected genes in the cotton brassinosteroid mutant *Pag1* [20] compared to wild type cotton. Interestingly, the results of qRT-PCR analyses indicated that all tested genes were up-regulated and had high mRNA levels in all tissues examined including hypocotyls, stems and 10 and 20 DPA fibers compared to WT plants. The relative expression of all 12 of these genes was much higher in leaves of the WT compared to the mutant (Figure 6A). These results may help to explain why all *GhPERK* genes did not show high expression levels during fiber development and in the other tested tissues in the WT plants because *Pag1* mutant plants display short stature with no obvious fiber development. The *GhPERK* genes could be responsible for this altered morphology through brassinosteroid catabolism but a more extensive investigation may be needed to explore the complex gene regulation. These results demonstrate the negative regulation of *GhPERK* genes in the brassinosteroid pathway.

In addition, to determine whether *GhPERK* gene expression can be regulated by brassinosteroids, we used qRT-PCR to examine the expression levels of *GhPERK* genes in cotton seedlings treated with the active form of brassinosteroid BL (brassinolide) and two brassinosteroid biosynthesis inhibitors, BRZ (brassinozole) and PCZ (propiconazole), for 0.5, 1, 3 and 5 hours (Figure 6B). Interestingly, all *GhPERK* genes were found to be regulated by BL, BRZ and PCZ treatments with no specific patterns. *GhPERK27* and *GhPERK23* expression was up-regulated by BL but was down-regulated by BRZ and PCZ at different time points in the experiment. However, *GhPERK5* expression was down-regulated at all time points for all treatments. In addition, none of the genes showed patterns of expression that were indicative of complex regulation. Taking all the results together, we hypothesize that the *GhPERK* genes are regulated by BR and play roles in the BR signaling pathway.

**Figure 7:**
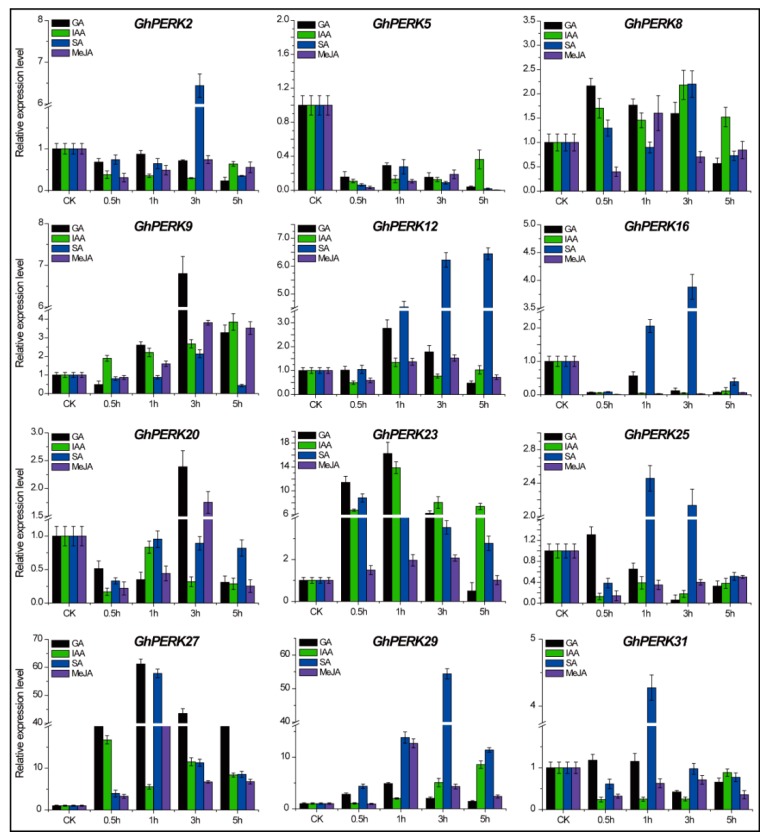
Expression pattern analysis of *GhPERK* genes under different phytohormonal stresses including GA, IAA, SA and MeJA in cotton seedlings by qRT-PCR analysis. Error bars indicated standard deviations (SD) among three independent biological experiments.

It has been reported previously that the expression of *Arabidopsis PERK4* responds to abscisic acid (ABA) during root tip growth [16,17]. In addition to the many phytohormone responsive *cis*-elements identified in this study, we evaluated the *GhPERK* genes for their responses to phytohormones including GA, IAA, SA and MeJA after 0.5, 1, 3 and 5 hours of treatment (Figure 7). Here, we also observed differential gene expression for the various genes in response to the different hormones over the 5-hour course of the experiment. *GhPERK8*, *GhPERK9*, *GhPERK12*, *GhPERK23*, *GhPERK27* and *GhPERK29* expression was mostly up regulated with a few exceptions. However, *GhPERK5* was the one gene that was down-regulated when the plants were exposed to all of the hormones at all time points. Moreover, the *GhPERK* genes displayed no specific expression patterns for any of the hormones, suggesting that the *GhPERK* genes experienced strong functional divergence following gene duplication during evolution.

### 2.7. Regulatory Sub-Networks Involving GhPERK and Other G. hirsutum Genes

**Figure 8:**
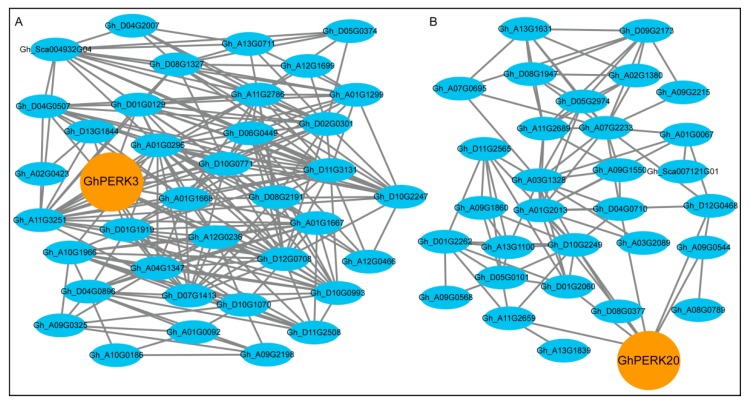
Co-expression analysis of *GhPERK* genes visualized by Cytoscape software. (**A**) Co-expression sub-network for *GhPERK3* gene. (**B**) Co-expression sub-network for *GhPERK20* gene. Nodes are connected by lines to show the interaction of that gene to other co-expressed gene.

Co-expressed genes show similar increases or decreases in transcription levels in different samples [44]. The knowledge about neo- or sub-functionalization of gene paralogues needs to study gene modules along with molecular evolutionary approaches. Co-expression networks are conserved across species and even across distinct kingdoms of life clearly indicating cross-kingdom orthologs presence. Co-expression networks have emerged as an important tool to rapidly infer the functional relatedness among genes belonging to similar functions/pathways [29]. The *PERKs* are known to influence several developmental, signaling and physiological processes in plants. The co-expression between *GhPERKs* and other *G. hirsutum* genes were analyzed using ccNET and co-expression genes were identified. We found that two *GhPERK* genes were co-expressed with other *G. hirsutum* genes. *GhPERK3* was co-expressed with 37 *G. hirsutum* genes (Figure 8A), while *GhPERK20* had 30 co-expressed *G. hirsutum* genes (Figure 8B). Further, Gene Ontology (GO) showed that these genes were enriched in 17 GO terms (FDR < 0.05) related to enzyme inhibitor activity, polygalacturonase activity, glycoprotein binding, calcium ion binding, actin binding, zinc ion binding and defense response (Table S6). Similarly, Kyoto Encyclopedia of Genes and Genomes (KEGG) enrichment analysis indicated that most of co-expressed genes were involved in plant-pathogen interaction, pentose and glucuronate interconversions, regulation of actin cytoskeleton, nitrogen metabolism, monoterpenoid biosynthesis and microbial metabolism in diverse environments (Table S6).

## 3. Discussion

Several previous studies explored the molecular and physiological functions of *PERK* genes in various plant species [1,14,15,16,17] but no studies describing the *PERK* genes and their functions in cotton have been published at present. Cotton is the largest and most important source of fiber for textile industry [30]. Ours the first ever study to identify the *PERK* genes in diploid and allotetraploid species of cotton and to analyze their evolutionary relationships, gene duplication and selection pressure and expression in response to various abiotic stresses and hormone treatments. Recent advances in cotton genome sequencing and genomics made it possible to conduct a comprehensive study of cotton *PERK* genes and investigate their potential functions. Our research identified and analyzed *PERK* genes in *G. arboreum, G. hirsutum* and *G. raimondii* but we mainly focused on the genes in *G. hirsutum*. The results of our study will provide basic information and serve as an important resource for further investigations into *PERK* gene function in cotton.

### 3.1. GhPERK Genes Show Evolutionary Conservation

In the present study, we classified PERK protein family members from *S. moellendorffii* (lycophytes), *P. taeda* (gymnosperm), *A. comosus* (angiosperm), *C. reinhardtii* (chlorophyte), *P. patens* (bryophytes), seven dicots (*A. thaliana, G. arboreum, G. hirsutum, G. raimondii, G. max, P. trichocarpa* and *T. cacao*) and three monocots (*O. sativa, S. bicolor* and *Z. mays*) into four phylogenetic clades. The phylogenetic analysis showed that the PERK proteins from *S. moellendorffii*, *A. comosus and* *P. taeda* divided the PERK proteins into these four clades. The phylostratum analysis of *PERK* gene family identified the earliest plant lineage as *PERK* genes were present in *C. reinhardtii* (chlorophyte), indicated that *PERK* genes were originated from early land plants phylostratum and potential orthologous genes of *PERK* are present throughout pant kingdom. Phylogenetic tree showed that PERK-b clade is an ancient group having *PERK* gene family members from all selected plant species except *P. taeda*. While, PERK-d clade could be more advance than others, lacking *PERK* genes from *S. moellendorffii*, *P. taeda*, *C. reinhardtii* and *P. patens*. The presence of *PERK* genes in each selected plant species, with 33 *PERK* genes from *G. hirsutum* and only five *PERK* genes from *P. patens*, demonstrated that *PERK* genes are evolutionary conserved and experienced a large scale expansion in higher plants. Moreover, cotton and cacao *PERK* genes depicted close relationship during phylogenetic analysis strengthening the previous finding that cotton and cacao share a common ancestor [25]. Sequence logos for conserved amino acid residues were highly conserved in *Arabidopsis*, rice and *G. hirsutum* at both the N and C termini and there was no compositional bias in any conserved amino acid residues indicating that the *PERK* genes are evolutionarily conserved which will help to identify the pattern of PERK protein sequence conservation in other plant species. The *WOX* and *YABBY* gene families were also found to be evolutionarily conserved and exhibit similar conserved sequence logos [31,45].

The promoter sequences of the *GhPERK* genes contain various predicted cis-elements that are specific for plant growth, development and light and stress responses. Transcription is regulated by the binding of transcription factors to upstream *cis*-acting regulatory elements. Many studies have shown the important role that light plays in the processes of plant growth and development [46]. A number of *cis*-elements such as the TCA and CGTCA elements for MeJA and SA stress, respectively [32,47], dehydration-response elements [48], abscisic acid (ABA) [49], heat stress response element [50], and the W-box for ABA and drought stress [51] were predicted in many of the *GhPERK* gene promoter regions. In our study, most of *GhPERK* genes have features typical of genes involved in growth and the responses to various stresses. For example, *AtPERK4* is involved in ABA-responsive root-tip growth in *Arabidopsis* [16,17]. Previously, gene families in cotton and other plants were found to have similar *cis*-elements in their promoters and were shown to be functionally relevant [52,53].

In our results, the uneven distribution of *GhPERK* genes on the At and Dt chromosomes of *G. hirsutum* indicates possible gene loss or addition through segmental or whole genome duplication events and incomplete genome sequencing could be an issue here as well. In addition, all *GhPERK* genes have no introns and the proteins have similar patterns of protein motif distribution. Previous studies have demonstrated the importance of introns in the evolution of various plant genes [33]. Many gene families indicate less or lack of introns in their gene families [34,52,54]. These exon/intron structural differences might be due to insertion/deletion events and are helpful in the identification of evolutionary mechanisms [55]. Introns are considered to be under weak selection pressure and the absence of introns indicates that *GhPERK* genes might have evolved relatively rapidly. Furthermore, the number of introns present early in gene family evolution were reduced over time, resulting in gene families with fewer or no introns [56] and these families are considered to be evolutionarily advanced [35], while gene families with more or larger introns are thought to have gained new functions during evolution.

It has been established that tandem duplication might cause an increase in intron numbers and create new genes [57]. Consistent with our findings, we did not identify and tandem duplication event in this study. Exon/intron structural conservation is functionally important and despite low levels of sequence conservation, the exon/intron structures in many gene families are conserved [58]. We speculate that the *GhPERK* gene family is an advanced gene family in which the members lost their introns over time but underwent expansion in a rapid phase of evolution.

### 3.2. GhPERK Gene Family Duplication and Expansion

*G. hirsutum* is an allotetraploid cotton species that is cultivated globally and accounts for 95% of global production [59]. The A- and D-genome cottons (*G. arboreum* and *G. raimondii*, respectively) experienced whole genome duplication and, as the result of hybridization, the allotetraploid AtDt genome species *G. hirsutum* emerged between 5 and 10 mya (million years ago) [41]. As allotetraploid crop, *G. hirsutum* is one of the best model species in which to study polyploidy in plants [28]. Gene duplication and divergence mainly lead toward evolutionary momentum [36]. Gene duplication generates functional divergence, which is essential for environmental adaptability and speciation [37]. Duplication is indicated when aligned sequences share more than 70% identity and cover more than 80% of the total length [38]. Two duplicated genes positioned on the same chromosome might be the result of tandem duplication, while their presence on different chromosomes of the same sub-genome is referred to as segmental duplication [60]. In segmental duplications, the duplicated genes are usually scattered throughout the genome, while tandemly duplicated genes tend to cluster together [61]. Approximately 65 mya, segmental along with whole genome duplications in ancestral plants contributed to the expansion of many gene families [62,63]. During evolution, two large scale segmental as well as small scale tandem duplications generated new genes that contributed genomic complexity to the plant kingdom [64].

In the present we study identified 33 *GhPERK* genes, double the number of *PERK* genes found in any other plant species, which demonstrates the effect of polyploidy on the *PERK* gene family in *G. hirsutum*. Polyploidy is a feature of genome evolution in *G. hirsutum* that consequently doubled the size of the *PERK* family as a result of segmental and whole genome duplication (WGD). As previously reported, polyploidy enabled many plant species to adapt to new environments [65]. The dramatic increase in the size of the *GhPERK* gene family can be evaluated by examining the *PERK* genes in the A- (*G. arboreum*) and D-genome (*G. raimondii*) cottons. Gene loss is an essential process that occurs after the hybridization event during genomic arrangements and chromosome doubling [26]. Even though all the other species included in our study also have many duplicated gene pairs, our phylogenetic analysis indicates that expansion of the *PERK* gene family in plants could be the result of segmental and whole genome duplication. Moreover, the *G. hirsutum* genome experienced fewer arrangements compared to paleopolyploid maize and *Brassica* [66,67].

Generally, polyploidy is associated with gene duplication and in our study we found that segmental and whole genome duplication were the main factors responsible for doubling the number of *GhPERK* genes. Segmental duplication is the main impetus during evolution and it has occurred in many plant genomes that contain many duplicated chromosomal blocks [64]. For example, many *Arabidopsis* gene families experienced coherent evolutionary dynamics that led to gene family expansion [68,69]. Furthermore, many gene families such as cotton *WOX*, *YABBY*, *GRAS*, *RH2FE3*, *MIKC*-Type and *MADS*-Box, soybean *WRKY* and sesame heat shock proteins experienced expansion as the result of segmental and whole genome duplication [31,45,52,54,70,71,72]. Our results showed that many of the *GhPERK* genes clustered into pairs, which indicates an ancient genome duplication event. The two copies of a gene can be subjected to shuffling and rearrangements that create potential diversity. Four possible fates have been described for duplicated genes [73]. One, one gene copy might be deleted during the evolutionary process, thereby removing functional redundancy. Two, sub-functionalization of both genes that shared the parental functions, leading to the development of partially different functions over time. Three, neo-functionalization in which one gene copy acquired new functions during evolution. Four, an intermediate form of sub- and neo-functionalization in which only genes critical for plant growth and development are retained. Thus, the large numbers of *PERK* genes in the *G. hirsutum* genome may be important for the plant’s normal growth and development and in responding to phytohormone and abiotic stresses. In our study, we found that the *GhPERK* genes experienced strong purifying selection.

*Arabidopsis* experienced whole genome duplication twice in the *Brassicaceae* lineage [74]. In Addition, cotton and cacao share a common ancestor that experienced an ancient duplication ~18-58 mya, eventually nascent duplication event in cotton again [25]. Orthologous/paralogous and gene duplication analysis showed that A-genome and At sub-genome *PERK* genes share a common ancestor, while D-genome and Dt sub-genome *PERK* genes belong to the same ancestor. Our investigations into cotton *PERK* gene family expansion provides useful information on chromosomal interaction and polyploidization in addition to differential rates of evolution and information transfer through inter-genomic hereditary. Taking all of our data together, we conclude that the cotton *PERK* gene family experienced segmental and whole genome duplication, which subsequently caused expansion of the *GhPERK* gene family.

### 3.3. GhPERK Genes are Regulated by Abiotic and Hormonal Stresses

A limited number of studies have been conducted to investigate the molecular and physiological functions of PERK proteins in plants. Previously, it has been reported that receptor-like protein kinases (RLKs) are likely to respond to external challenges in an-ever changing environment and are involved in hormonal response pathways, plant growth and development, self-incompatibility, cell differentiation, symbiosis and pathogen recognition [1]. In *Arabidopsis*, *PERK1* was shown to be induced rapidly in response to wounding [14]. *PERK4* a novel regulator of Ca^2+^ signaling, mediates the early events of the abscisic acid response in root tip growth [16,17]. However, no systematic study of PERK genes in cotton has been published at present.

The results of our study show that all predicted *GhPERK* genes are highly expressed in leaves and some stages of ovule development, demonstrating the positive roles that these genes may play in cotton leaf morphology with no obvious contribution to fiber development, implying a negative role in fiber development. Also, the expression of *GhPERK* genes can be significantly regulated (positively or negatively) by abiotic stress treatments such as cold, heat, salt, and PEG because all of the *GhPERK* genes displayed ubiquitous expression patterns that did not follow any particular fashion. Our qRT-PCR results agreed with previously published RNA-seq and transcriptomic data. Coupled with these findings, we conclude that *GhPERK* genes may provide important genetic strategies for breeding cotton lines that are tolerant to several abiotic stresses.

Further experiments indicated that the *GhPERK* genes could play a role in negative regulation of the brassinosteroid signaling pathway; the expression of all genes was up-regulated in all examined tissues in the cotton brassinosteroid mutant *Pag1* compared to WT plants. Moreover, the expression of all tested *GhPERK* genes could be either up- or down-regulated by the active form of brassinosteroid, BL and by treatment with the two brassinosteroid biosynthesis inhibitors BRZ and PCZ. These findings show that further investigations are needed to explore the functions of *GhPERK* genes in the brassinosteroid signaling pathway. Additionally, all *GhPERK* genes showed significant responses upon exposure to several phytohormones including GA, IAA, SA and MeJA; the expression of *GhPERK8*, *GhPERK9*, *GhPERK12*, *GhPERK23*, *GhPERK27* and *GhPERK29* was up-regulated with a few exceptions, while *GhPERK5* was down-regulated when the plants were exposed to all four plant hormones at all time points from 0.5 to 5 hours, supporting our hypothesis that *GhPERK* genes play essential roles in certain hormone signaling pathways. We can also conclude that the *GhPERK* genes experienced strong functional divergence following gene duplication during the evolution of the *GhPERK* gene family in cotton.

### 3.4. GhPERK co-expressed Genes Showed Functional Divergance

Proline-rich extensin-like receptor kinases (*PERKs*) are an important class of receptor kinases. These are associated with multiple molecular and cellular mechanisms affecting developmental, signaling and physiological processes in plants [15]. In the current study, a co-expressed network indicated that two *PERK* genes were co-expressed with one or more other *G. hirsutum* genes. Gene Ontology (GO) showed that these genes were related to enzyme inhibitor activity (GO:0004857), polygalacturonase activity (GO:0004650), glycoprotein binding (GO:0005515), calcium ion binding (GO:0005509), actin binding (GO:0003779), zinc ion binding (GO:0008270) and defense response (GO:0006952). Additionally, a KEGG enrichment analysis revealed that these two *PERK* genes could be involved in plant-pathogen interaction (K02183), pentose and glucuronate interconversions (K01051), regulation of actin cytoskeleton (K05765), nitrogen metabolism (K01674), monoterpenoid biosynthesis (K07385) and microbial metabolism in diverse environments (K00860). These results indicated that *PERK* genes could be involved in several molecular, biological and physiological processes that might be the result of *PERK* genes functional divergence during long evolutionary history of cotton.

## 4. Materials and Methods

### 4.1. Identification of PERK Protein Family Members

The *PERK* genes and encoded proteins in other species were identified via a BLAST search using the *Arabidopsis PERK* genes [15] and encoded protein sequences as queries in *G. arboreum* (ICR, version 1.0), *G. hirsutum* (NAU, version 1.1), *G. raimondii* (JGI, version 2.0), *G. max* (version 10), *P. trichocarpa* (version 2.0), *T. cacao* (version 10), *O. sativa* (version 10), *Z. mays* (version 10), *S. bicolor* (version 10), *A. comosus* (version 3.0), *C. reinhardtii* (version 5.5), *P. patens* (version 3.3), *S. moellendorffii* (version 1.0) and *P. taeda* (version 1.0). Moreover, the definition of the PERK (PF07714) domain was downloaded from Pfam: http://pfam.janelia.org/ [75] and then the hidden Markov model (HMM) was used to verify the *PERKs* from other species. The *G. arboreum* database was downloaded from (ftp://bioinfo.ayit.edu.cn/downloads/) while *G. raimondii* and *G. hirsutum* were downloaded from COTTONGEN (https://www.cottongen.org/). Also, *Arabidopsis* database was downloaded from TAIR 10 (http://www.Arabidopsis.org), while sequence databases for the other plant species were downloaded from online Phytozome v11 (https://phytozome.jgi.doe.gov/pz/portal.html). All retrieved sequences were verified by SMART (http://smart.embl-heidelberg.de/) [76] and Interproscan 63.0 (http://www.ebi.ac.uk/InterProScan/) [77]. The biophysical properties were predicted with the ExPASy ProtParam tool (http://us.expasy.org/tools/protparam.html) and subcellular localization was predicted using softberry (www.softberry.com).

### 4.2. Sequence Logos and Phylogenetic Analysis

Sequence logos for conserved amino acid residues and phylogenetic analysis were performed as described previously [45]. In brief, the sequence logos were constructed using online tool WEBLOG [39], while the phylogenetic tree was generated with MEGA 7.0 [78] using the Maximum likelihood (ML) method. Bootstrapping with 1000 replicates was used to test the statistical support of the nodes in the phylogenetic trees. The default parameters were used to generate the sequence logos and phylogenetic trees used in this study.

### 4.3. Exon/Intron, Protein Motifs and Promoter cis-elements Analysis

To determine exon/intron structure, bed-files obtained from databases were analyzed using GSDS 2.0 (http://gsds.cbi.pku.edu.cn/index.php) [79] and the NJ tree was constructed with MEGA 7.0 as described above. Protein motif distribution patterns were determined using the online MEME tool (http://meme-suite.org/tools/meme) [80] as described [81]. For *cis*-element analysis in promoter regions, 2-kb upstream sequences were analyzed in PlantCARE [82] and the elements were categorized based on their predicted functions.

### 4.4. Genomic Distribution, Gene Duplication and Synteny Analysis

To determine the chromosomal distribution of *GhPERK* genes, extracted gff3-files of cotton genome annotation data (ftp://ftp.bioinfo.wsu.edu/species/Gossypium_hirsutum/ NAU-NBI_G) were used to map the genes to the chromosomes using MapInspect (http://www.plantbreeding.wur.nl/UK/software_mapinspect.html) [83]. Gene duplication analysis and Ka/Ks values were calculated using methods described previously [45]. In short, CIRCOS [84] was used to generate the figure and Ka/Ks values were calculated using PAL2NAL (http://www.bork.embl.de/pal2nal/) [85] and the CODEML program in the PAML package [86].

### 4.5. Expression Analysis, Stress Treatments, qRT-PCR and Co-expression Network Analysis

In our study, the *G. hirsutum* variety CRI24 and the cotton brassinosteroid mutant *Pag1* were used for gene expression analysis. To determine the tissue-specific expression patterns, all sampled tissues were obtained from cotton plants grown under field conditions with standard cultural practices. Abiotic stress treatments were conducted as described previously [31,54]. For hormonal stresses, the seedlings were treated with BL (10 µM), BRZ (10 µM), PCZ (10 µM), GA (100 µM), IAA (100 µM), SA (10 µM) and MeJA (10 µM) for 0.5, 1, 3 and 5 h. Seedlings collected at 0 hour were used as the control (CK). All samples were immediately frozen in liquid nitrogen and kept at −80 °C prior to use.

Total RNA was extracted from cotton samples using the RNAprep Pure Plant Kit (Tiangen, Beijing, China) and cDNA was synthesized from 1 μg total RNA using the Prime-Script® RT reagent kit (Takara, Dalian, China). The *GhHis3* gene (GenBank accession no. AF024716) was used as the internal control for normalization of gene expression and the qRT-PCR assays were performed with SYBR Green on a LightCycler 480 system (Roche Diagnostics GmbH, Sandhofer Straße 116, 68305 Mannheim, Germany). All the primers used in this study are given in Table S7. The relative expression values were calculated using the method described previously [87]. Co-expression data for *GhPERK* genes was downloaded from online tool ccNET [88] (http://structuralbiology.cau.edu.cn/gossypium/cytoscape/network.list.php) and finally, we visualized co-expression network using Cytoscape (version 3.4.0) program [89]. Additionally, the putative functions of co-expressed genes based on Gene Ontology (GO) and Kyoto Encyclopedia of Genes and Genomes (KEGG) enrichment analyses were investigated from online tool CottonFGD (http://www.cottonfgd.org/analyze/).

## 5. Conclusions

The present study identified 207 *PERK* gene family members in 15 different plant species. Among these genes, we identified 33 *GhPERK* genes from the allotetraploid cotton species *G. hirsutum*. Sequence analysis of conserved amino acid residues among the PERK proteins from *Arabidopsis*, rice and *G. hirsutum* showed that *PERK* genes are highly conserved in plants. Phylogenetic analysis grouped all PERK proteins into four clades on the basis of sequence homology. *GhPERK* genes do not have introns and the protein motif distribution pattern is conserved across all proteins. Furthermore, the predicted *cis*-elements in the *GhPERK* gene promoter regions indicate their functional relevance to growth, development and the response to various stresses. Uneven distribution of the *GhPERK* genes on the At and Dt chromosomes of *G. hirsutum* may be due to gene loss or addition through segmental or whole genome duplication during evolution, and/or incomplete genome sequencing. Also, gene duplication and synteny analysis indicates that the cotton *PERK* genes experienced segmental and whole genome duplication during evolution that subsequently resulted in a major expansion of the *GhPERK* gene family. Additionally, our data shows that the *GhPERK* genes experienced functional divergence during evolution and their high levels of expression in leaves can be regulated by abiotic and hormonal stresses, indicating that members of this gene family could play crucial roles in hormone signaling pathways, especially for the brassinosteroids. Moreover, *PERK* genes could be involved in several molecular, biological and physiological processes that might be the result of *PERK* genes functional divergence during long evolutionary history of cotton.

## Supplementary Materials

The following are available online at https://www.mdpi.com/1422-0067/20/7/1750/s1. Figure S1. Phylogenetic analysis and tree was generated by NJ method among cotton *PERK* genes including *G. arboreum, G. hirsutum* and *G. raimondii* in order to estimate common ancestor hypothesis. Tree divided all *PERK* genes into six groups represented by different colors. Figure S2. Exon/intron and protein motif distribution of *GhPERK* genes couples with phylogenetic tree constructed by NJ method. (A) Exon/intron distribution of *GhPERK* genes. (B) Protein motif distribution of *GhPERK* genes. Figure S3. Chromosomal distribution of *GhPERK* genes on their corresponding chromosomes. A01 to A13 and D01 to D13 represents At and Dt sub-genomes *G. hirsutum* chromosomes, respectively. Chromosomal sizes were calculated from published genome data. Table S1. Gene locus ID and their proposed names of all observed species including *A. thaliana, G. arboreum, G. hirsutum, G. max, G. raimondii, O. sativa, P. trichocarpa, S. bicolor*, *, T. cacao* and *Z. mays.* Table S2. Biophysical properties of *GhPERK* genes including locus ID, start and end point, strand, CDs (coding sequence), protein length, MW (molecular weight), pl (isoelectric point), gravity and predicted subcellular localization. Table S3. Promoter *cis*-element prediction of *GhPERK* genes was characterized according to their relatedness to growth and development, light and different stresses. Table S4. Orthologous/paralogous in At, Dt sub-genomes of *G. hirsutum*, *G. arboreum* (A genome) and *G. raimondii* (D genome). Ka/Ks (non-synonymous/synonymous) values of all identified orthologous/paralogous gene pairs were also mentioned. Table S5. RNA-seq data analysis of *GhPERK* genes in fuzzless/lintless mutants (*M1l* and *M2l*). Additionally, genes were classified depending on their up or down regulated expression pattern. Table S6. Gene Ontology (GO) and Kyoto Encyclopedia of Genes and Genomes (KEGG) enrichment analysis of other *G. hirsutum* co-expressed genes with *GhPERKs.* Table S7. List of all oligonucleotide primers used in this study.

## Abbreviations

NaCl	Sodium chloride
PEG	Polyethylene glycol
GA	Gibberellic acid
IAA	Indole-3-acetic acid
BL	Brassinolide
BRZ	Brassinazole
PCZ	Propiconazole
MeJA	Methyl jasmonate
SA	Salicylic acid
DPA	Days post-anthesis
µM	Micro-molar
Mya	Million years ago
*G. arboreum*	*Gossypium arboreum*
*G. hirsutum*	*Gossypium hirsutum*
*G. raimondii*	*Gossypium raimondii*
*Z. mays*	*Zea mays*
*O. sativa*	*Oryza sativa*
*G. max*	*Glycine max*
*A. thaliana*	*Arabidopsis thaliana*
*P. trichocarpa*	*Populus trichocarpa*
*T. cacao*	*Theobroma cacao*
*S. bicolor*	*Sorghum bicolor*
