# Supplementary figures and images for: Genome-Wide Identification and Characterization of the PERK Gene Family in Gossypium hirsutum Reveals Gene Duplication and Functional Divergence
Source: Int J Mol Sci. 2019 Apr 9;20(7):1750. doi: 10.3390/ijms20071750 (PMC6479967; doi:10.3390/ijms20071750)

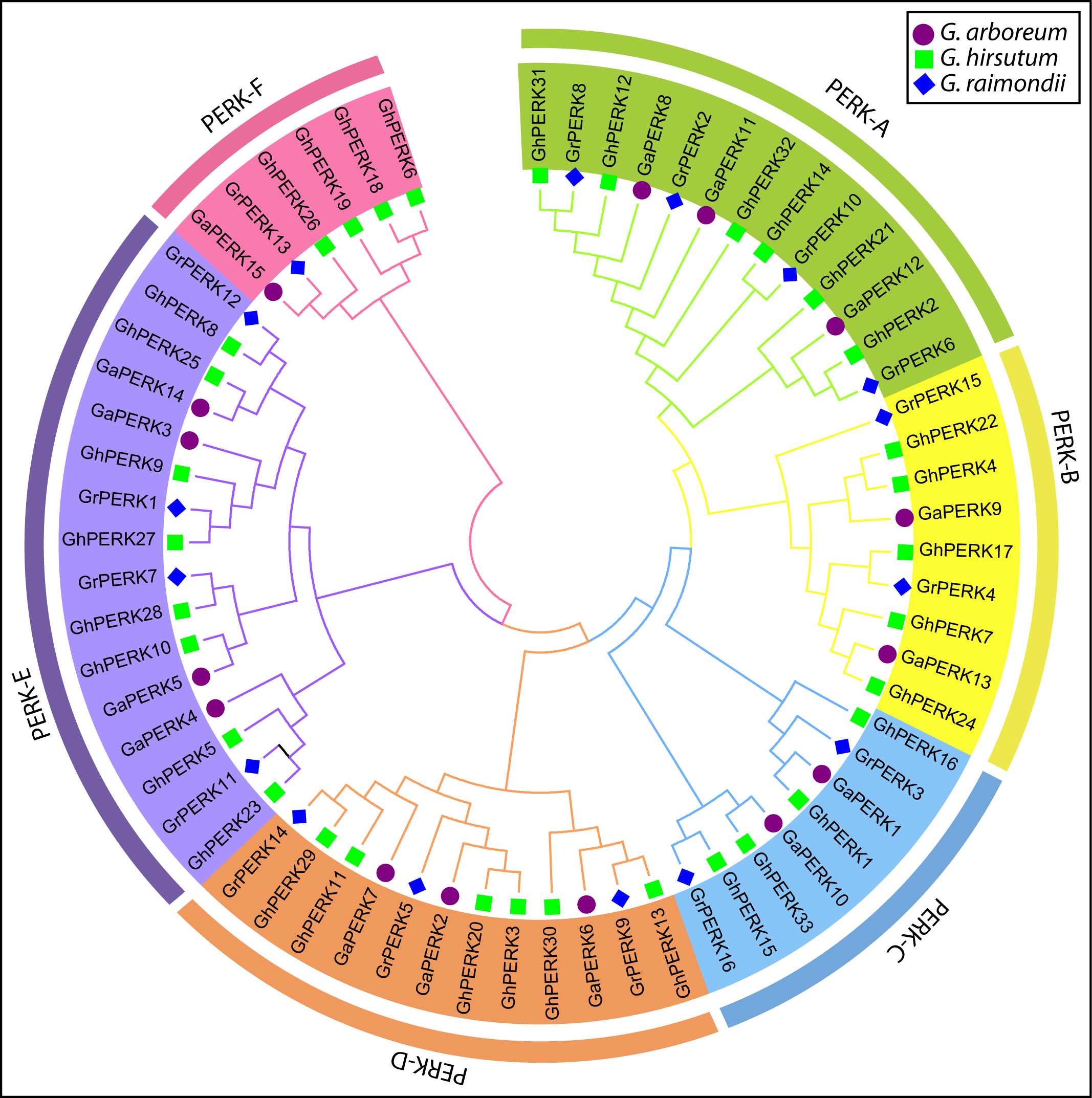

Supplement: Supplementary file 1 [file ijms-20-01750-s001.zip › Figure. S1.tif]

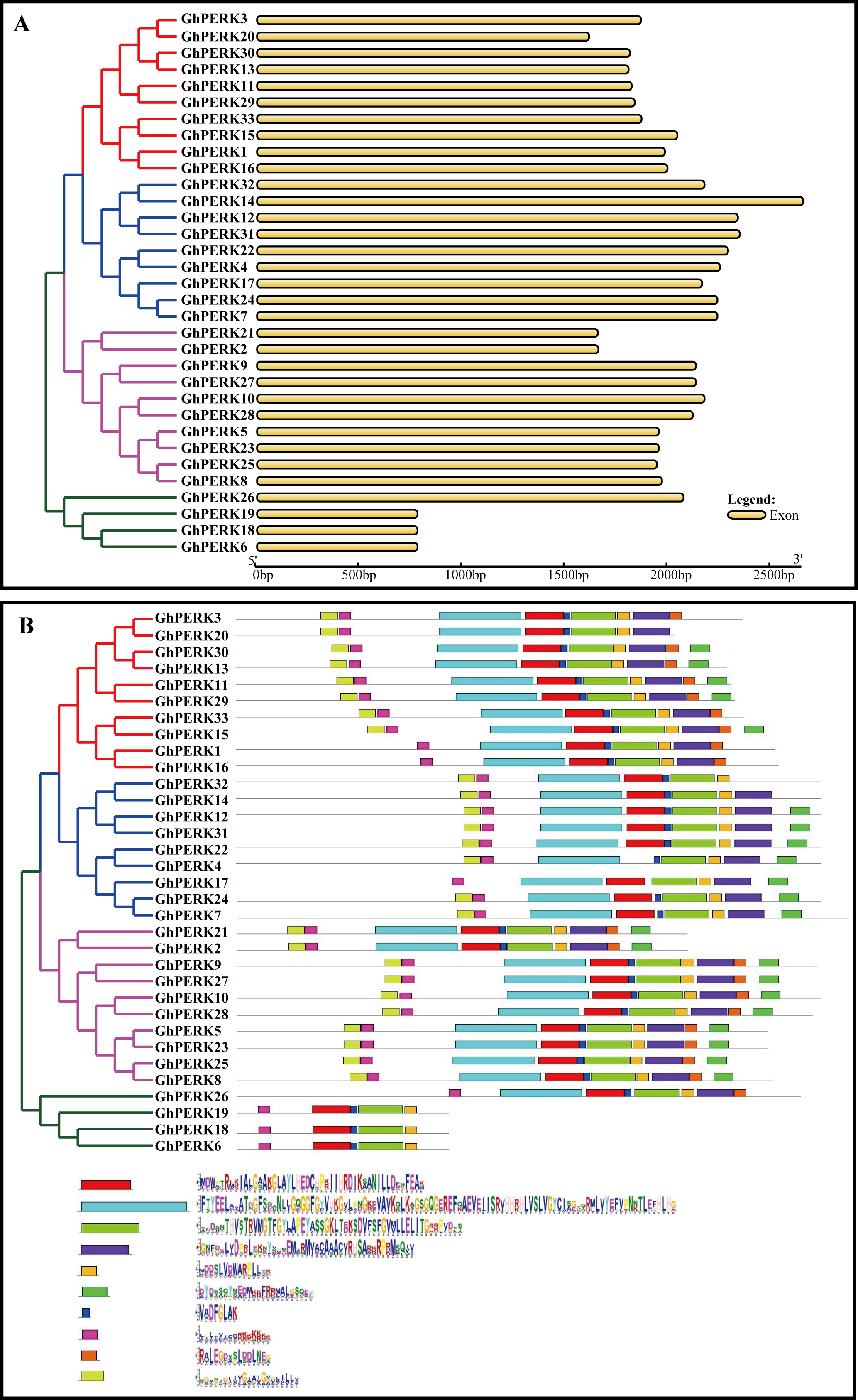

Supplement: Supplementary file 1 [file ijms-20-01750-s001.zip › Figure. S2.tif]

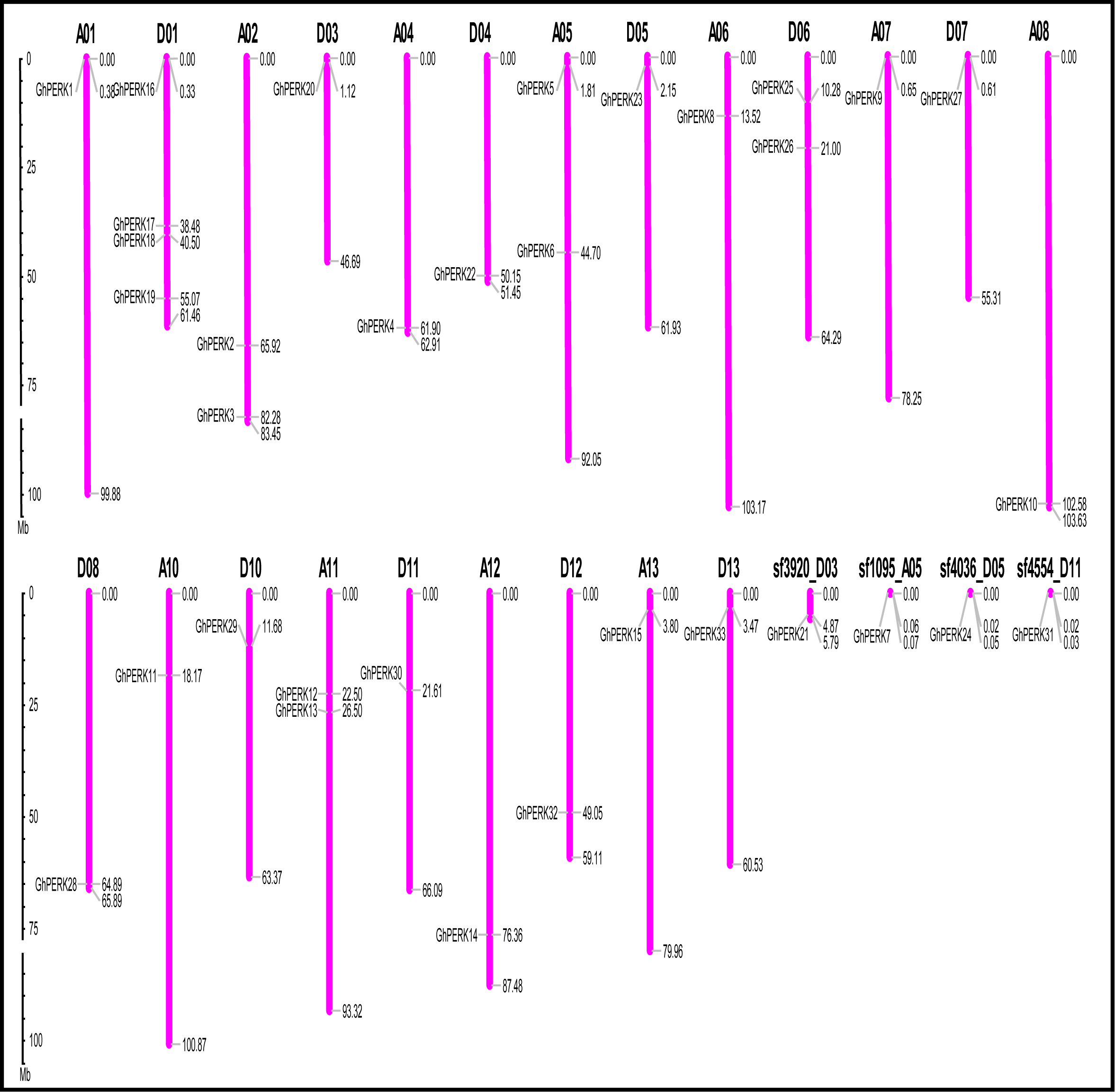

Supplement: Supplementary file 1 [file ijms-20-01750-s001.zip › Figure. S3.tif]
